# Telocytes in pancreas of the Chinese giant salamander (*Andrias davidianus*)

**DOI:** 10.1111/jcmm.12948

**Published:** 2016-09-20

**Authors:** Hui Zhang, Pengcheng Yu, Shengwei Zhong, Tingting Ge, Shasha Peng, Xiaoquan Guo, Zuohong Zhou

**Affiliations:** ^1^ College of Animal Science and TechnologyJiangxi Agricultural UniversityNanchangChina

**Keywords:** amphibian, pancreas, telocytes, ultrastructure

## Abstract

Telocytes (TCs), novel interstitial cells, have been identified in various organs of many mammals. However, information about TCs of lower animals remains rare. Herein, pancreatic TCs of the Chinese giant salamanders (*Andrias davidianus*) were identified by CD34 immunohistochemistry (IHC) and transmission electron microscopy (TEM). The IHC micrographs revealed CD34^+^ TCs with long telopodes (Tps) that were located in the interstitium of the pancreas. CD34^+^ TCs/Tps were frequently observed between exocrine acinar cells and were close to blood vessels. The TEM micrographs also showed the existence of TCs in the interstitium of the pancreas. TCs had distinctive ultrastructural features, such as one to three very long and thin Tps with podoms and podomers, caveolae, dichotomous branching, neighbouring exosomes and vesicles. The Tps and exosomes were found in close proximity to exocrine acinar cells and α cells. It is suggested that TCs may play a role in the regeneration of acinar cells and α cells. In conclusion, our results demonstrated the presence of TCs in the pancreas of the Chinese giant salamander. This finding will assist us in a better understanding of TCs functions in the amphibian pancreas.

Telocytes (TCs) have been confirmed as a novel type of interstitial/stromal cell 1, 2. TCs were first identified in 2005 and their name was coined in 2010 by Popescu and his team 3, 4. Morphologically, the key characteristic of the TCs when compared with other interstitial cell types is the presence of one to five very thin and long cell body prolongations, called telopodes (Tps) (10–1000 μm), with alternating regions of podoms (˜0.5 μm) and podomers (˜0.1 μm), visible with electron microscopy 1, 2, 5. Transmission electron microscopy (TEM) has been essential in the identification and recognition of TCs/Tps according to their distinctive ultrastructural characteristics 6. Functionally, TCs in tissues can form a complex 3D interstitial network by their long Tps, establishing homocellular contacts between Tps and heterocellular contacts with other nearby cell types, including myocytes, stem/progenitor cells, immunoreactive cells and nerves 7. Furthermore, TCs/Tps can release extracellular vesicles loaded with microRNAs to regulate neighbouring cell functions 8. TCs are suggested to play critical roles in tissue support, intercellular signalling, cell differentiation, immune surveillance, stem/progenitor cells guidance and nurse, tissue repair and regeneration and cell expansion and movement 1, 9, 10, 11, 12.

In the last 10 years, TCs have been identified in many organs of various animals, especially in mammals, including human, pig, gerbil, mouse, rat, degu and canine 1, 2, 4, 13, 14, 15, 16, 17. There are also a few studies regarding TCs in lower animals, such as the newt, zebrafish, turtle and chicken 2, 18, 19, 20. TCs have become an important research focus in cell biology and translational medicine. In comparison with mammals, however, there are few investigations of TCs in lower animals. Our previous study has demonstrated the existence of TCs in the ileum of the Chinese giant salamander *Andrias davidianus* (Amphibia: Caudata) 21. To determine whether TCs are also present in other organs of the amphibian, further studies are required. In this study, we identified TCs in the pancreas of the Chinese giant salamander by IHC and TEM.

This work was approved by the Ethical Committee for Animal Care and Use of Jiangxi Agricultural University after relevant ethical review according to the National Institutes of Health Guide for the Care and Use of Laboratory Animals. The 2.5‐year‐old Chinese giant salamanders (two males and two females; weight: 0.99–1.12 kg) were raised with simulated ecological breeding technology in their primary habitat provided by an artificial breeding farm in the mountain area of Jiangxi province, China. After putting on the ice, the Chinese giant salamanders were killed and the pancreases were quickly excised and fixed in 4% paraformaldehyde and 2.5% glutaraldehyde/phosphate‐buffered saline (PBS) (pH 7.4, 0.1 M) respectively. The immunohistochemistry (IHC) was performed according to the protocol provided by the anti‐CD34 antibody manufacturer (Abcam, Cambridge, UK). The 3,3‐diaminobenzidine (DAB) was used as the chromogen. The sections were then counterstained with haematoxylin and mounted. Finally, the sections were photographed by BM 2000 light microscopy (Yongxin, Nanjing, China). TEM was conducted according to the methods of Yang *et al*. 20 and Zhang *et al*. 22. Briefly, small pieces of pancreas tissue were fixed in 2.5% glutaraldehyde/PBS (pH 7.4, 0.1 M) at 4°C for 72 hrs, and then they were fixed later in 1% OsO_4_ (Polysciences Inc., Warrington, PA, USA) for 1 hr, dehydrated in a concentration series of ethanol, infiltrated with propylene oxide/araldite mixture and embedded in araldite. After the samples were sectioned, the sections were stained with 1% uranyl acetate and Reynold's lead citrate for 20 min. The stained sections were observed and photographed by a high‐resolution digital camera connected to the TEM (Hitachi H‐7650, Tokyo, Japan). The long and short diameters of the TC cell bodies, the width of the podoms and podomers of the Tps and the length of the Tps were measured in the IHC and TEM images by Image Pro‐plus 7.0 software (Media Cybernetics, Rockville, MD, USA). Additionally, the diameters of secretory granules of the α cells and zymogen granules of exocrine acinar cells were also measured. Data were analysed by Excel software (Microsoft, Redmond, WA, USA).

The cell bodies and Tps of CD34 immune‐positive (CD34^+^) TCs were stained brown in the Chinese giant salamander pancreas (Fig. 1). The CD34^+^ TCs nuclei were immune negative and stained blue as a result of the haematoxylin counterstaining. The pancreatic exocrine acinar cells were negative, cone‐shaped and contained round nuclei in their basal portions. The apical portions of the exocrine acinar cells had many small, round and foam‐type zymogen granules. The CD34^+^ TCs were frequently observed in the interstitium of the pancreas (Fig. 1) and were located in the connective tissues among acini and adjacent to pancreatic exocrine acinar cells. Moreover, the CD34^+^ TCs were frequently observed in close proximity to the blood vessels (Fig. 1). The cell bodies of CD34^+^ TCs were pyriform, triangular or spindle shaped. A characteristic feature of the CD34^+^ TCs was the presence of one to three very long Tps (Fig. 1). The Tps frequently extended into connective tissues among the spherical acini. There were also some Tps extended between two acinar cells, and even traversing the entire spherical acinus (Fig. 1). The podoms and podomers of Tps were also clearly observed (Fig. 1). The average long and short diameters of cellular bodies of Tps, and the average length of CD34^+^ TCs, as measured on IHC images, were 17.29 ± 4.55 μm, 9.46 ± 1.32 μm and 25.91 ± 10.80 μm respectively. In addition, the blood cells were also CD34^+^, but the vascular endothelia were negative (Fig. 1).

**Figure 1:**
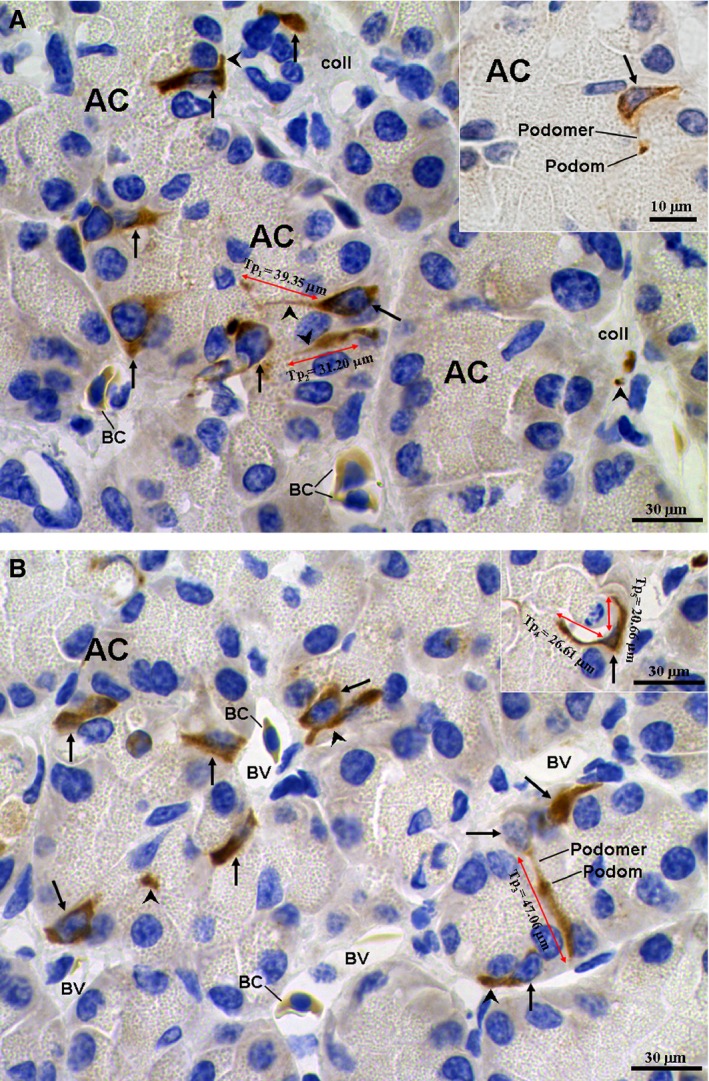
CD34 immunohistochemistry photomicrographs of the pancreas. (**A**) Brown CD34^+^ telocytes (TCs; black arrows) are shown. The blood cells are also CD34^+^. The CD34^+^ TCs with long telopodes (Tps; black arrowheads) are located in the stroma between exocrine acinar cells (AC). TCs/Tps are frequently observed adjacent to the blood vessels (BV). Inset: a spindle‐shaped TC with a Tp containing podomer and podom. (**B**) CD34^+^ TCs are found in the pancreas. A TC with a long Tp_3_ traverses the entire spherical acinus. The podoms and podomers of the Tp_3_ were also clearly observed. Inset: a triangulate TC with two Tps (Tp_4_ and Tp_5_) surrounding an AC. BC, blood cells; coll, collagen fibres.

TCs and their Tps were also observed in the interstitium among the pancreatic exocrine acini as shown in the TEM micrographs (Figs. 2, 3, 4, 5, 6). The cell bodies of the TCs appeared piriform and contained a big nucleus, which was surrounded by a small amount of cytoplasm (Figs. 2 and 3). TCs usually possessed one to three very long and thin Tps. In particular, an extremely long Tp was measured as 77.03 μm (Fig. 3A). The Tps had some distinctive ultrastructural features, including podoms and podomers (Figs. 2A, 4 and 5), dichotomous branching patterns (Figs. 3 and 5) and caveolae (Figs. 2B and 5). Moreover, the exosomes (Figs. 2B and 3B) and big membranous vesicles (Fig. 5) were found in the vicinity of the Tps. The mean width of podoms and podomers, as measured on TEM images, were 0.71 ± 0.16 μm and 0.22 ± 0.05 μm respectively. The Tps were frequently observed adjacent to exocrine acinar cells (Figs. 2A, 3, 4 and 5), which contained many round and electron‐dense zymogen granules. The average diameter of zymogen granules was 2.16 ± 0.29 μm. In addition, the Tps were also located adjacent to an endocrine α cell (Fig. 6), which contained many small, denser and electron‐dense secretory granules in their cytoplasm. The average diameter of secretory granules of α cell was 0.39 ± 0.05 μm, which was less than that of the zymogen granules of exocrine acinar cells. The endocrine α cell was surrounded by cytoplasmic processes of exocrine acinar cells (Fig. 6).

**Figure 2:**
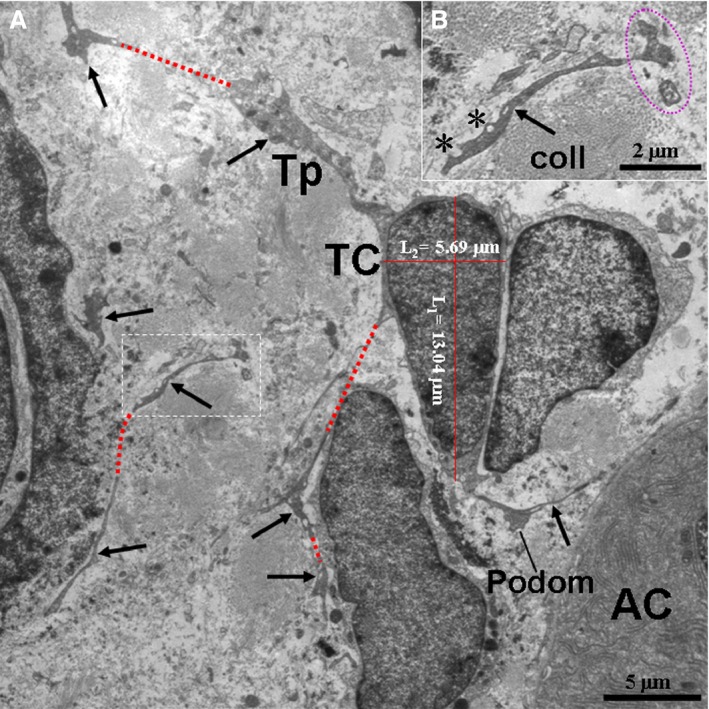
TEM micrographs of the pancreas of the Chinese giant salamander. (**A**) The pyriform telocytes (TCs) with telopodes (Tps; black arrows) are present in the stroma of the pancreas. (**B**) High‐magnification TEM micrograph of the dashed line boxed areas shown in (**A**) with details of a Tp. The red dashed lines show possible sinuous Tp segments. The purple outlines show the exosomes. The asterisks show the caveolae. AC, exocrine acinar cells; Tp, telopode; coll, collagen fibres.

**Figure 3:**
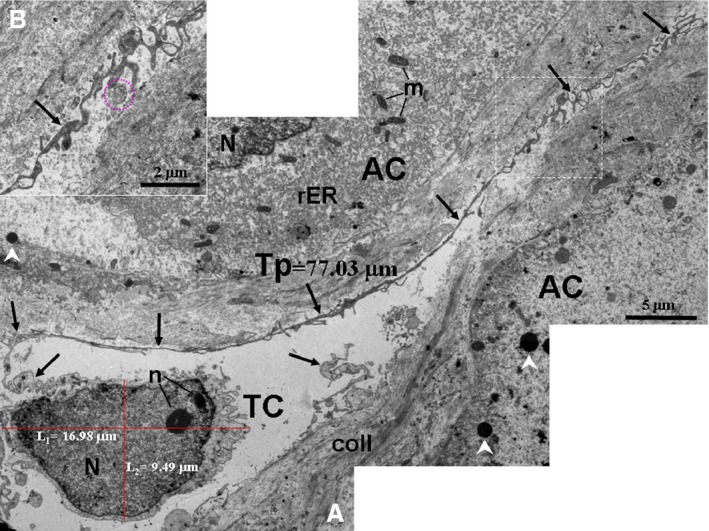
TEM micrographs of the pancreas of the Chinese giant salamander. (**A**) A telocyte (TC) with an extremely long telopode (Tp) (77.03 μm) (black arrows) is present between two exocrine acinar cells (AC). (**B**) High‐magnification TEM micrograph of the dashed line boxed areas shown in (**A**) with details of a Tp. The Tp exhibits dichotomous branching. The white arrowheads show zymogen granules of AC. The purple outlines show the exosomes. coll, collagen fibres; N, nucleus; n, nucleolus; rER, rough endoplasmic reticulum; m, mitochondrion.

**Figure 4:**
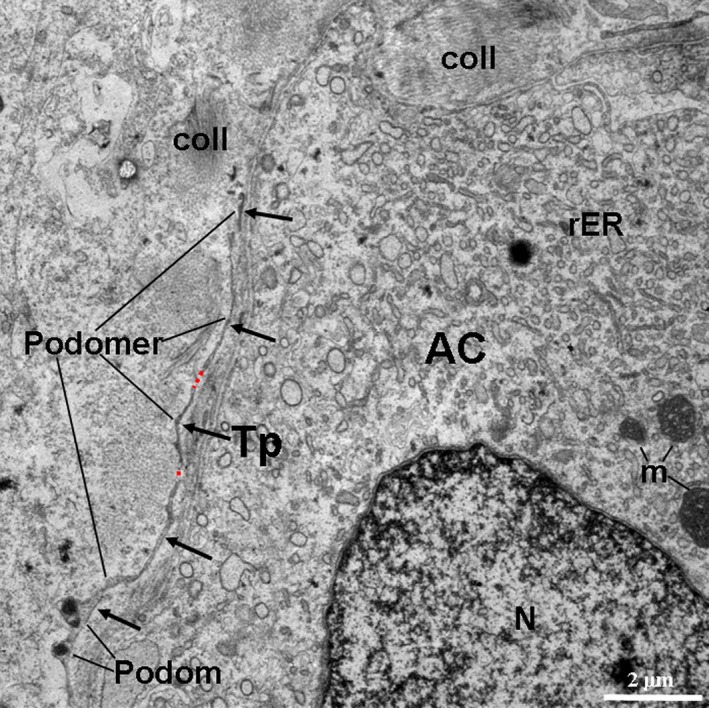
TEM micrograph of the pancreas of the Chinese giant salamander. A telopode (Tp; black arrows) is located in close proximity to an exocrine acinar cell (AC). The thick podoms and thin podomers are also observed. The red dashed lines show possible sinuous segments of Tps. coll, collagen fibres; N, nucleus; rER, rough endoplasmic reticulum; m, mitochondrion.

**Figure 5:**
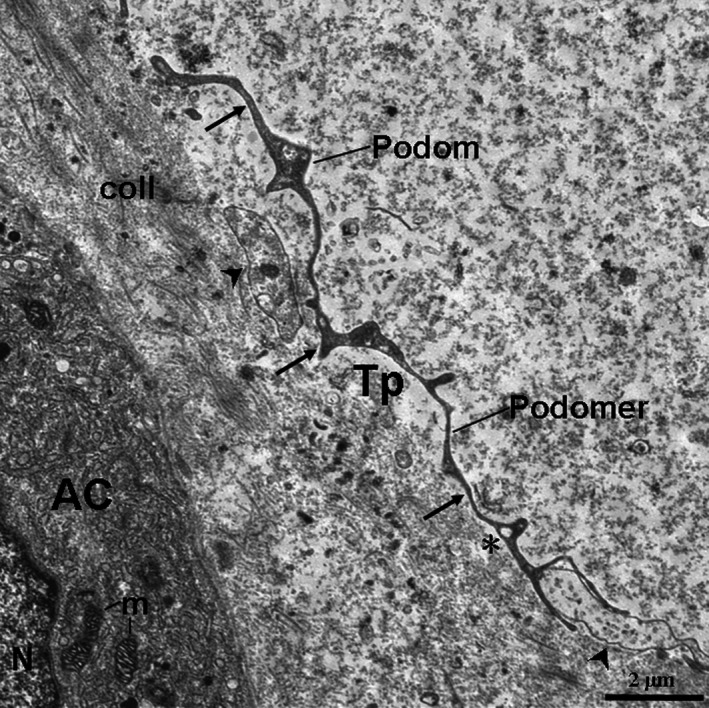
TEM micrographs of the pancreas of the Chinese giant salamander. A telopode (Tp; black arrows) is located adjacent to an exocrine acinar cell (AC). Two big membranous vesicles (black arrowheads) are found in the vicinity of the Tp. The asterisk shows the caveolae. coll, collagen fibres; N, nucleus; m, mitochondrion.

**Figure 6:**
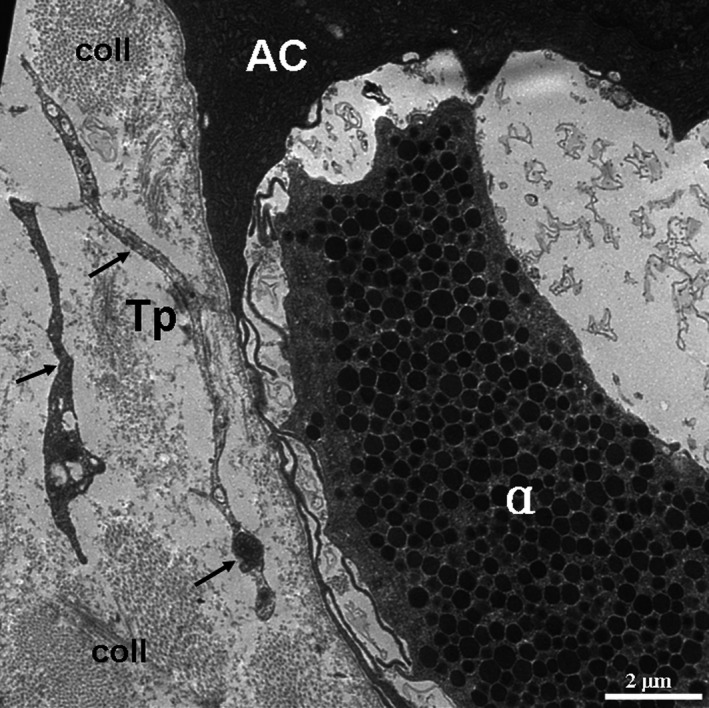
TEM micrographs of the pancreas of the Chinese giant salamander. Two telopodes (Tps; black arrows) are present in close proximity to an α cell, which is encapsulated by the cytoplasmic processes of an exocrine acinar cells (AC). The α cell has many small, round, electron‐dense secretory granules. α, α cell; coll, collagen fibres; Tp, telopode.

Previous investigations have documented that TCs are present in the pancreas of several mammals, such as human, rat, cat and degu 13, 23, 24, 25. In this study, we demonstrated the presence of TCs in the pancreas of a lower animal, the Chinese giant salamander, by IHC and TEM according to the diagnostic criteria for TCs recommended by Popescu *et al*. 4, 25. TCs of the amphibian had a classical ultrastructure similar to mammals. A difference between the amphibian and mammals TCs is that the Tps of the amphibian TCs are not convoluted like mammalian Tps, though they do have dichotomous branching 4. The presence of TCs in the amphibian pancreas suggests that TCs might exist widely in animal pancreases of different evolutionary status. However, currently, the roles of TCs in tissue have not been thoroughly determined. Generally, TCs are believed to control growth and differentiation of other cell types, mainly stem/progenitor cells, and to further regulate neighbouring cell regeneration and tissue repair 1, 11, 26. Previous studies of the mammalian pancreas have demonstrated that TCs are mainly located in the stroma 23, 25. There are also pancreatic TCs between the acinar cells 24. TCs/Tps establish close spatial relationships with capillaries, acinar cells, stellate cells, mast cells, macrophages, nerve fibres and pancreatic ducts 23, 25. Based on the structural characteristics of TCs and their locations in other cell types, TCs could play a role in intercellular signalling 25. They may also act as pacemaker cells for the spontaneous rhythmic pancreatic duct contractions 24. In this study, the TCs/Tps were observed adjacent to pancreatic exocrine acinar cells. Moreover, the exosomes were also found between Tps and acinar cells. These results suggest that TCs and exocrine acinar cells of the amphibian pancreas may be functionally related. Previous studies also demonstrated that TCs were often observed adjacent to exocrine acinar cells, ducts, nerves and blood vessels in the pancreas 13, 23, 24, 25. However, knowledge of the structural correlations of TCs and endocrine cells in the pancreas remain absent. In this study, the Tps were observed in close proximity to a type of endocrine cell, the α cell. Accordingly, TCs may also function on endocrine cells, as well as play the role of TCs in exocrine acinar cells. In conclusion, our results confirm the existence of TCs in the pancreas of an amphibian. This information will help us to better understand the roles of TCs in the amphibian pancreas.

## Conflicts of interest

The authors declare that there are no conflicts of interest.
